# Renal capsular invasion is a prognostic biomarker in localized clear cell renal cell carcinoma

**DOI:** 10.1038/s41598-017-18466-9

**Published:** 2018-01-09

**Authors:** U-Syn Ha, Kyu Won Lee, Jin-hyung Jung, Seok-Soo Byun, Cheol Kwak, Jinsoo Chung, Eu Chang Hwang, Yong-June Kim, Tae Gyun Kwon, Seok Ho Kang, Sung-Hoo Hong

**Affiliations:** 1 Department of Urology, College of Medicine, The Catholic University of Korea, Seoul, Republic of Korea; 2 Biostatistics, Biomedicine & Health Sciences, College of Medicine, The Catholic University of Korea, Seoul, Republic of Korea; 3 Department of Urology, Seoul National University Bundang Hospital, Seongnam, Republic of Korea; 4 Department of Urology, Seoul National University College of Medicine, Seoul, Korea; 5 Department of Urology, Center for Prostate Cancer, National Cancer Center, Goyang, Korea; 6 Department of Urology, Chonnam National University Medical School, Gwangju, Korea; 7 Department of Urology, Chungbuk National University College of Medicine, Cheongju, Korea; 8 Department of Urology, Kyungpook National University School of Medicine, Daegu, Korea; 9 Department of Urology, Korea University School of Medicine, Seoul, Korea

## Abstract

Renal capsular invasion (RCI) and lymphovascular invasion (LVI) are potential prognostic factors of significance in renal cell carcinoma (RCC). We evaluated the independent prognostic implications of RCI and LVI in localized clear cell RCC based on a large multi-institutional cohort. 6, 849 patients who had undergone radical or partial nephrectomy for RCC were included. Associations between recurrence and RCI or LVI were analyzed by constructing statistical models that combined Cox proportional hazard regression and propensity score matching. To analyze RCI, 2, 733 patients including 603 patients with RCI were enrolled. To analyze LVI, 3, 586 patients including 121 patients with LVI were enrolled. Recurrence was observed in 75 (12.4%) patients with RCI and 134 (6.3%) patients without RCI. In all statistical models, RCI was significantly associated with an increased risk of recurrence. Recurrence was observed 29 (24.0%) patients with LVI and 207 (6.0%) patients without LVI. LVI was significantly associated with an increased risk of recurrence only in non-adjusted univariate models, but not in multivariate adjusted analysis or propensity score matching models. In conclusion, these findings suggest that RCI could be a significant risk factor for localized clear cell RCC recurrence. In contrast to RCI, LVI cannot be an independent prognostic variable.

## Introduction

The prognosis of patients with renal cell carcinoma (RCC) is currently assessed by the TNM staging system after surgical treatment such as radical or partial nephrectomy. Currently, the TNM staging system in RCC has used tumor size as the single deciding factor for classifying T1-2 RCC. However, despite appropriate surgical treatment in localized RCC, some patients experience unexpected disease progression or recurrence^1^. Therefore, it is insufficient to predict prognosis based only on tumor size because all localized RCCs do not demonstrate the same biological behavior and postsurgical clinical course. Although tumor grade currently provides valuable prognostic information, additional reliable factors are needed to predict prognosis more accurately.

Renal capsular invasion (RCI) and lymphovascular invasion (LVI) are two potential prognostic factors of significance. There is conflicting information regarding the prognostic implications of RCI. Data presented by Klatte *et al*.^2^, May *et al*.^3^, and Cho *et al*.^4^ suggested that RCI is a poor prognostic factor, but Süer E *et al*.^5^ and Rouach *et al*.^6^ reported that RCI is not an independent prognostic factor for disease-specific survival.

Penetration and migration of tumors into the blood or lymphatic vessels could be an essential step in entering the circulation^7^. Although macroscopic invasion into the vessels has been recognized as a prognostic factor for quite a while^8^, the International Society of Urological Pathology recommended that microvascular invasion should not be used as a prognostic factor in routine evaluations based on the accumulated evidence^9^. However, LVI has been investigated as an independent predictor of poor prognosis in several solid tumors^10–12^ and predisposing patients to disease progression. Studies addressing the prognostic implications of LVI provided conflicting suggestions^13–21^ and did not reach solid conclusions.

Large cohort studies on LVI and RCI that exclude and control for potential bias are currently not available. Therefore, there is still some controversy regarding the prognostic significance of RCI and LVI in RCC. The main aim of the current study was to present the independent prognostic implications of RCI and LVI in localized clear cell RCC based on a large multi-institutional cohort study.

## Methods

### Study populations

The data for this study was derived from the KORCC (Korean Renal Cell Carcinoma) database, a nationwide multicenter database from 8 academic centers in Korea. In total, 6, 849 patients who had undergone surgical treatments for RCC between 1999 and 2011 were included in the KORCC database^22^. Clinical data were reviewed and collected in the form of a standardized electronic case report that included the following: (1) preoperative data, including age, gender, body mass index (BMI), previous medical history, Eastern Cooperative Oncology Group (ECOG) performance status, American Society of Anesthesiologists (ASA) status classification system, and symptoms at diagnosis; (2) preoperative laboratory findings; (3) surgical data, including the operative method, operative time, and estimated blood loss; (4) pathologic data, including TNM stage, histologic subtype, Fuhrman’s nuclear grade, mass size, sarcomatoid differentiation, renal capsule invasion and lymphovascular invasion; Pathology specimens were assessed by experienced pathologists at each institution without centralized review. RCI was defined as the presence of tumor cells within the fibrous renal capsule without perirenal fat tissue infiltration, and LVI was defined as the presence of tumor cells inside small blood vessels or lymphatic channels within the tumor (excluding the renal vein and its muscle-containing segmental branches); (5) postoperative follow-up data, including disease recurrence, death, and cause of death. Pathological staging was performed based on the 7th edition of the American Joint Committee on Cancer classification system^23^, and histological differentiation was classified following the AJCC and Heidelberg recommendations^24^. Information on deaths and their causes (‘cancer-related death’ or ‘non-cancer-related death’) was updated by reviewing medical records and the Korean National Statistical Office database. After approval by the Institutional Review Boards of the Catholic University of Korea, Seoul St. Mary’s Hospital, we reviewed data from 3, 576 patients with localized clear cell RCC treated with curative surgery who were eligible for analysis.

### Statistical Analysis

Differences in clinicopathologic features including age, gender, body mass index (BMI), comorbidities (diabetes, hypertension and chronic renal disease), ECOG performance status, tumor size and Fuhrman grade were evaluated by descriptive statistics. Continuous and categorical variables were described using the independent t-test and χ^2^ test, respectively. The primary oncologic outcome measured in this study is recurrence. Recurrence-free survival rates were calculated using the cumulative incidence method and analyzed using the Kaplan-Meier method. Associations between recurrence and RCI or LVI were analyzed by constructing statistical models that combined Cox proportional hazard regression and propensity score matching.

To account for inherent differences among patients, such as baseline characteristics or uneven pathologic distributions between the two groups, the estimated propensity score was obtained from the fit of a logistic regression model to adjust for age, sex, BMI, tumor size, Fuhrman grade, sarcomatoid differentiation, ECOG performance status, and smoking status (c-statistic of 0.862 for RCI and 0.862 for LVI). 1:5 matching was performed using the greedy matching method, and the balance of the patients according to RCI or LVI was evaluated using the standardized difference and significance testing (independent t-test and χ² test or Fisher’s exact test) after propensity score matching. All statistical analyses were performed using the software package SAS version 9.3 (SAS Institute, Inc, Cary, NC, USA). A value of p < 0.05 was considered statistically significant.

## Results

### Comparison of clinical characteristics

To evaluate the prognostic significance of RCI, 2, 733 patients including 603 patients with RCI (incidence of RCI was 22.1%) were enrolled. To analyze LVI, 3, 586 patients including 121 patients with LVI (incidence of LVI was 3.4%) were enrolled. RCI and LVI were significantly associated with several other adverse clinicopathologic features, such as Fuhrman grade, and histology type (Tables 1 and 2).

**Table 1:** Baseline description of renal cell carcinoma patients with or without capsular invasion according to pre- and post-propensity matching.

	Pre-propensity cohort			Post-propensity cohort		
	RCI (−)	RCI (+)	*P* value	RCI (−)	RCI (+)	*P* value
Age (years)	55.5 ± 12.6	57.2 ± 12.4	0.0032	55 ± 13	56.6 ± 12.6	0.0795
BMI	24.6 ± 3.3	24.5 ± 3.1	0.3677	24.7 ± 3.3	24.9 ± 3	0.3214
Sex			0.9549			0.0584
Male	1532 (71.92)	433 (71.81)		838 (73.19)	169 (73.8)	
Female	598 (28.08)	170 (28.19)		307 (26.81)	60 (26.2)	
DM			0.1511			0.0584
No	1668 (83.86)	438 (81.26)		961 (84.15)	181 (79.04)	
Yes	321 (16.14)	101 (18.74)		181 (15.85)	48 (20.96)	
HTN			0.1616			0.476
No	1218 (62.02)	314 (58.69)		716 (63.2)	139 (60.7)	
Yes	746 (37.98)	221 (41.31)		417 (36.8)	90 (39.3)	
CKD			0.3148			0.1759
No	1937 (98.27)	533 (98.89)		1117 (98.41)	228 (99.56)	
Yes	34 (1.73)	6 (1.11)		18 (1.59)	1 (0.44)	
Smoking status			0.0653			0.0791
Non-smoker	1016 (60.23)	243 (59.41)		629 (56.92)	135 (59.47)	
Ex-smoker	301 (17.84)	91 (22.25)		205 (18.55)	51 (22.47)	
Current smoker	370 (21.93)	75 (18.34)		271 (24.52)	41 (18.06)	
ECOG_index			0.0987			0.794
0	1815 (86.59)	510 (85.57)		983 (87.53)	200 (88.5)	
1	259 (12.36)	73 (12.25)		124 (11.04)	24 (10.62)	
2	22 (1.05)	13 (2.18)		16 (1.42)	2 (0.88)	
Fuhrman grade			<0.0001			0.8018
1 & 2	1347 (63.9)	316 (52.93)		703 (61.4)	139 (60.7)	
3	706 (33.49)	233 (39.03)		424 (37.03)	85 (37.12)	
4	55 (2.61)	48 (8.04)		18 (1.57)	5 (2.18)	
Sarcomatoid differentiation			<0.0001			0.7765
No	1387 (98.86)	332 (94.05)		1137 (99.3)	227 (99.13)	
Yes	16 (1.14)	21 (5.95)		8 (0.7)	2 (0.87)	
Tumor size	31.4 (30.5–32.3)	43.3 (41.1–45.7)	<0.0001	30.9 (29.9–32)	32 (29.9–34.3)	0.4049

**Table 2:** Baseline description of renal cell carcinoma patients with or without lymphovascular.

	Pre-propensity cohort			Post-propensity cohort		
	LVI (−)	LVI (+)	*P* value	LVI (−)	LVI (+)	*P* value
Age (years)	56 ± 12.6	58 ± 12.1	0.0885	56.5 ± 12.8	59.2 ± 11.6	0.1064
BMI	24.6 ± 3.3	24.3 ± 3.2	0.3293	24.6 ± 3.5	24.5 ± 3.3	0.7841
Sex			0.7192			0.595
Male	2486 (71.75)	85 (70.25)		246 (71.3)	47 (68.12)	
Female	979 (28.25)	36 (29.75)		99 (28.7)	22 (31.88)	
DM			0.8164			0.6425
No	2667 (83.95)	100 (84.75)		281 (81.45)	57 (83.82)	
Yes	510 (16.05)	18 (15.25)		64 (18.55)	11 (16.18)	
HTN			0.0903			0.1687
No	1889 (60.03)	80 (67.8)		206 (60.23)	47 (69.12)	
Yes	1258 (39.97)	38 (32.2)		136 (39.77)	21 (30.88)	
CKD			0.0871			0.2068
No	2476 (97.56)	117 (100)		335 (97.67)	67 (100)	
Yes	62 (2.44)	0 (0)		8 (2.33)	0 (0)	
Smoking status			0.0359			0.6264
Non-smoker	1742 (65.64)	45 (52.94)		190 (56.38)	34 (50)	
Ex-smoker	377 (14.2)	19 (22.35)		70 (20.77)	16 (23.53)	
Current smoker	535 (20.16)	21 (24.71)		77 (22.85)	18 (26.47)	
ECOG_index			0.0027			0.8813
0	2299 (72.5)	89 (87.25)		286 (84.37)	59 (86.76)	
1	576 (18.16)	11 (10.78)		47 (13.86)	8 (11.76)	
>=2	296 (9.33)	2 (1.96)		6 (1.77)	1 (1.47)	
Fuhrman grade			<0.0001			0.4718
1 & 2	2038 (62.21)	37 (34.58)		120 (34.78)	21 (30.43)	
3	1123 (34.28)	58 (54.21)		187 (54.2)	37 (53.62)	
4	115 (3.51)	12 (11.21)		38 (11.01)	11 (15.94)	
Sarcomatoid differentiation			0.0081			0.5178
No	2358 (98.54)	71 (94.67)		331 (95.94)	65 (94.2)	
Yes	35 (1.46)	4 (5.33)		14 (4.06)	4 (5.8)	
Tumor size	33.1 (32.2–34)	59 (53.7–64.9)	<0.0001	58.8 (56.1–61.7)	58.7 (52.8–65.3)	0.9812

Invasion according to pre- and post-propensity matching.

In the RCI group, 120 patients were matched with 600 patients without RCI. In the LVI group, 61 patients were matched with 305 patients without LVI. The median follow-up durations in patients with RCI and without RCI were 39 (interquartile range [IQR]: 16–72) and 31 (IQR: 12–60) months, respectively (Tables 1 and 2). The median follow-up durations in patients with LVI and without LVI were 37 (IQR: 13–68) and 34 (IQR: 11–62) months, respectively.

### Impact of RCI on oncological outcomes

**Figure 1:**
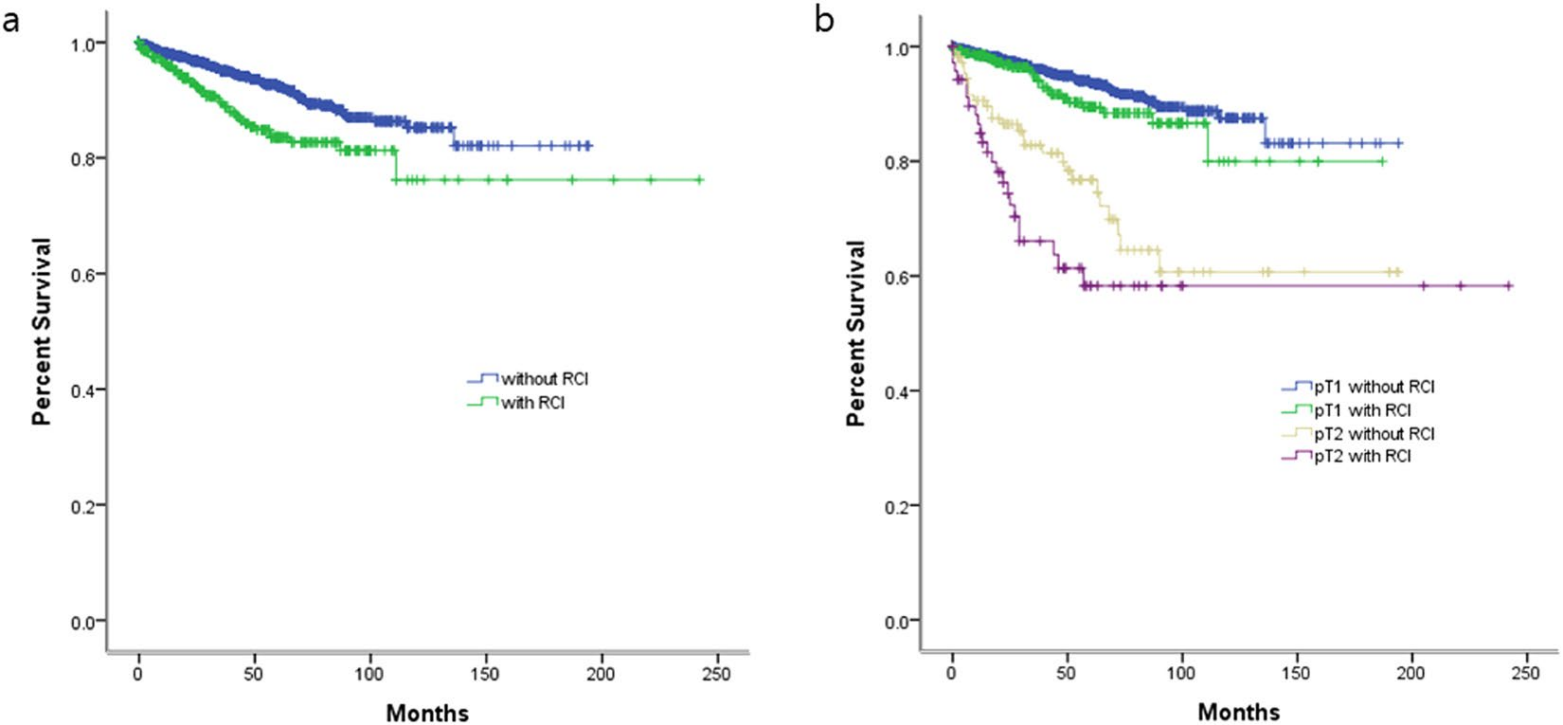
(**a**) Kaplan-Meier survival curve of localized RCC according to renal capsular invasion (RCI). Recurrence-free survival rate of patients with RCI and without RCI were 83.5% and 92.4% (p < 0.001, log rank test). (**b**) Patients with RCI in pT1 and pT2 were correlated with cancer recurrence, respectively (p < 0.001, p = 0.088, log rank test).

Among all patients evaluated for RCI, recurrence was observed in 209 (7.6%) total patients including 75 (12.4%) patients with RCI and 134 (6.3%) patients without RCI. The 5-year recurrence-free survival rates were 83.5% and 92.4% in patients with and without RCI, respectively (log rank test, p < 0.001, Fig. 1a. When patients were stratified into 4 groups based on RCI and tumor stage (pT1 and pT2), the 5-year recurrence-free survival rates were 89.4% in pT1 patients with RCI versus 93.7% in those without RCI (log rank test, p < 0.001) and 58.3% in pT2 patients with RCI versus 76.7% those without RCI (log rank test, p = 0.088) (Fig. 1b).

In separate Cox hazards analysis for recurrence, a non-adjusted univariate (H.R: 2.154, C.I: 1.588–2.923; model 1), and a multivariate adjusted analysis (H.R: 1.668, C.I: 1.060–2.626; model 2), RCI was significantly associated with an increased risk of recurrence (Table 3). In the propensity score matching analysis, RCI was significantly associated with an increased risk of recurrence (H.R: 2.130, C.I: 1.201–3.777) in model 3, and this association remained significant (H.R: 2.057, C.I: 1.146–3.693) in model 4, which combined propensity score matching with adjusting for various potential prognostic factors (Table 3).

**Table 3:** Adjusted hazard ratios of disease recurrence according to analysis model.

		H.R	95% C.I	P-value
Renal capsule invasion	Model1^a^	2.154	1.588–2.923	<0.0001
	Model2^b^	1.668	1.060–2.626	0.027
	Model3^c^	2.130	1.201–3.777	0.0097
	Model4^d^	2.057	1.146–3.693	0.0156
Lymphovascular invasion	Model1^a^	3.642	2.438–5.440	<0.0001
	Model2^b^	1.687	0.938–3.034	0.0809
	Model3^c^	1.242	0.693–2.227	0.4676
	Model4^d^	1.285	0.709–2.328	0.4082

^a^Not adjusted.

^b^Adjusted for tumor size, Fuhrman grade, sarcomatoid differentiation, age, sex, DM, HTN, ECOG index, BMI, smoking status, and CKD.

^c^PS matching for tumor size, Fuhrman grade and sarcomatoid differentiation.

^d^PS matching and adjusted for age, sex, DM, HTN, ECOG index, BMI, smoking status, and CKD.

### Impact of LVI on oncological outcomes

**Figure 2:**
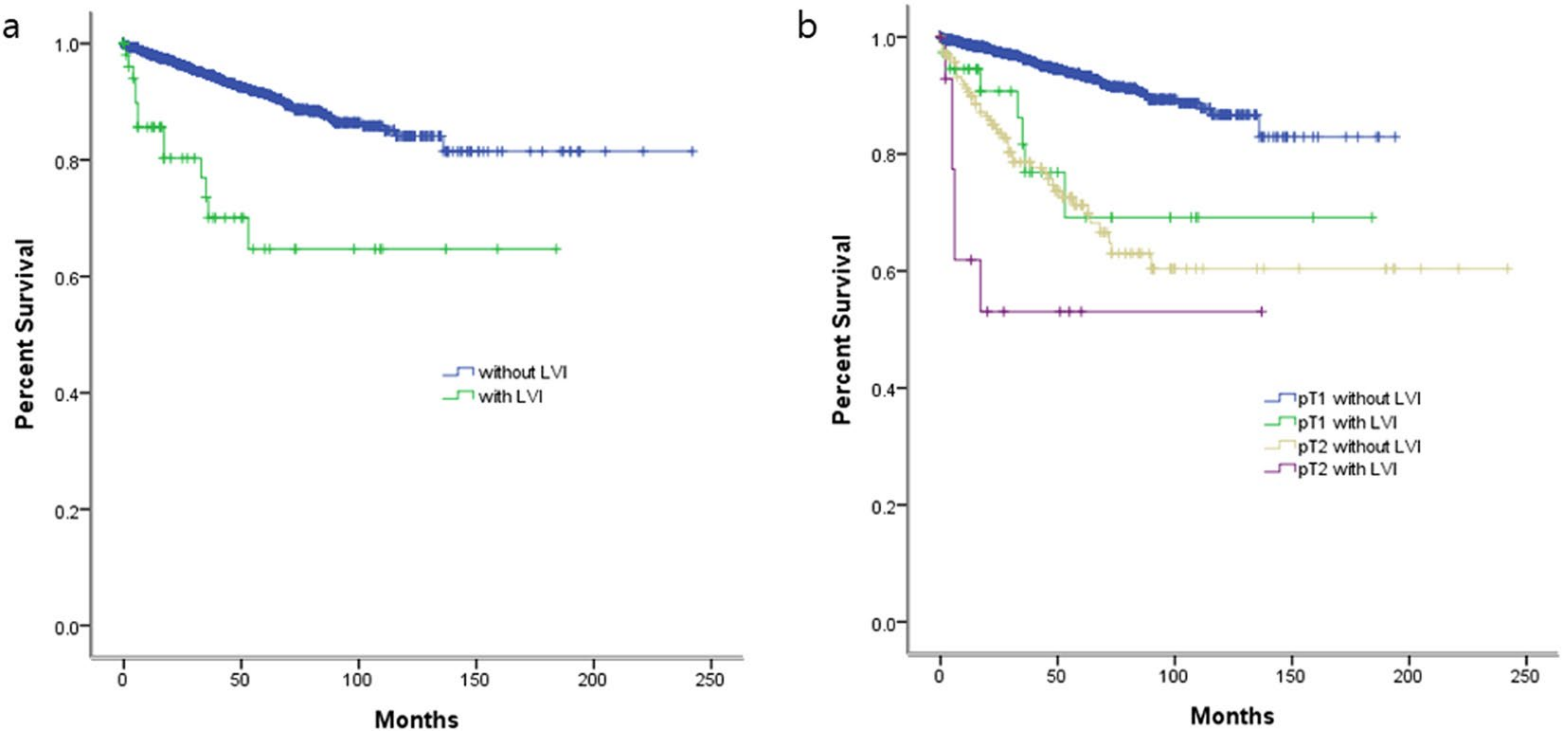
(**a**) Kaplan-Meier survival curve of localized RCC according to lymphovascular invasion (LVI). Recurrence-free survival rate of patients with LVI and without LVI were 64.7% and 91.3% (p < 0.001, log rank test). Patients without LVI were correlated with a higher recurrence-free survival rate (p < 0.001, log rank test). (**b**) Patients without RCI in pT1 and pT2 were correlated with cancer recurrence, respectively (p < 0.001, p = 0.026, log rank test).

When evaluating patients for LVI, recurrence was observed in 236 (15.2%) patients including 29 (24.0%) patients with LVI and 207 (6.0%) patients without LVI. The 5-year recurrence-free survival rates were 64.7% and 91.3% in patients with and without LVI, respectively (log rank test, p < 0.001, Fig. 2a). When the patients were stratified into 4 groups based on the LVI and stage (pT1 and pT2), the 5-year recurrence-free survival rates were 69.2% in pT1 patients with LVI versus 93.4% in those without LVI (log rank test, p < 0.001) and 53.1% in pT2 patients with LVI versus 71.3% those without LVI (log rank test, p = 0.026) (Fig. 2b).

In separate Cox hazards analysis for recurrence, LVI was significantly associated with an increased risk of recurrence only in non-adjusted univariate analysis (H.R: 3.642, C.I: 2.438–5.440; model 1), and not in multivariate adjusted analysis (H.R: 1.687, C.I: 0.938–3.034; model 2) (Table 3). In the PS matching analysis, LVI was not significantly associated with an increased risk of recurrence (H.R: 1.242, C.I: 0.69–2.227) in model 3, and this association remained nonsignificant (H.R: 1.285, C.I: 0.709–2.328) in model 4, which combined PS matching with adjusting for various potential prognostic factors (Table 3).

### Predictive factors for LVI

Table 4 shows the logistic regression model that predicted LVI in patients who underwent curative surgery for RCC. The odds ratio of LVI increased significantly with poor pathologic parameters. RCCs with large size, higher Fuhrman grade, sarcomatoid differentiation, and smoking were more likely to have LVI. High ECOG performance status score were inverse relation with LVI.

**Table 4:** Predictive factors for LVI in patients who underwent curative surgery for RCC.

	H.R. (95% CI)	p
Age	1.013 (0.998, 1.028)	0.0939
BMI	0.973 (0.918, 1.032)	0.3656
Sex		0.8344
Male	0.958 (0.643, 1.428)	
Female	1 (ref.)	
DM		0.5873
No	1 (ref.)	
Yes	0.866 (0.514, 1.457)	
HTN		0.0198
No	1 (ref.)	
Yes	0.611 (0.404, 0.925)	
CKD		0.2154
No	1 (ref.)	
Yes	0.17 (0.01, 2.802)	
Smoking status		0.022
Non-smoker	1 (ref.)	
Ex-smoker	1.884 (1.055, 3.367)	
Current smoker	2.131 (1.169, 3.886)	
ECOG_status		0.0044
0	1 (ref.)	
1	0.49 (0.26, 0.922)	
>= 2	0.159 (0.039, 0.651)	
Fuhrman grade		<0.0001
1 & 2	1 (ref.)	
3	2.859 (1.875, 4.359)	
4	5.646 (2.859, 11.146)	
Sarcomatoid differentiation		0.0149
No	1 (ref.)	
Yes	3.746 (1.294, 10.845)	
Tumor size (Per 1 cm)	5.332 (3.651, 7.787)	<0.0001

## Discussion

The main findings of this study for patients with localized clear cell RCC are as follows. (1) Patients with RCI were more likely to develop recurrence of RCC independent of confounding variables. RCI could be an important risk factor for recurrence in localized RCC. (2) The association of LVI with the risk of recurrence was significant only in univariate analysis. (3) After adjusting for confounding factors, LVI did not influence the recurrence of RCC following curative surgery.

The renal capsule is a tough fibrous layer surrounding the kidney and is covered in a thick layer of perinephric adipose tissue. These fibrous layers could provide protection from tumor seeding or spreading to adjacent tissue. Capsular invasion by the tumor would be early step to spread, which reflects tumor aggressiveness. Several studies have investigated the prognostic significance of RCI, but there are substantial inconsistencies regarding the association between RCI and oncological outcomes^2–6,25^. These studies had a relatively small sample size (the sample size in largest study was 653 patients), and the number of oncological events such as recurrence or death was small, in accordance with small cohort size. Therefore, the statistical value in previous studies is limited due to the small event number in a small cohort.

Moreover, many of these previous studies included various histological types of RCC such as clear cell, papillary, and chromophobe. The enrollment of patients with heterogeneous RCC subtypes could have resulted in bias and discordances^26^. The positive rate of RCI in previous studies that included heterogeneous histological types showed a wide range between 21.6% and 37.5%^2–6,25^ compared to the current study, which could also have affected the outcomes of the analysis. Therefore, the role of RCI as a prognostic factor remains controversial due to the small number of heterogeneous RCCs in the cohort.

To overcome the limitations of previous studies, the current study was conducted based on a large number of homogenous RCCs in the cohort, and demonstrated the significant impact of RCI on recurrence.

Campbell *et al*.^27^, found that RCI was detected more frequently in RCC patients with positive Cav-1 expression compared to those with negative Cav-1 expression. This study suggested that Caveolin-1 (Cav-1) promoted cell invasion in RCC cell lines and was a powerful predictor of metastasis in patients with clinically confirmed disease, which supports the relevance of RCI for prognosis in the current study by presenting a molecular biological mechanism.

LVI is thought to be associated with a predisposition toward recurrence or metastasis, because metastasis starts with malignant cells accessing the circulation through the blood or lymphatic vessels^7,28^. LVI is an important prognostic factor in other urinary tract malignancies such as bladder^29^ and upper urinary tract^10^ cancers. However, as in RCI, there are conflicting results regarding the prognostic significance of LVI for RCC. Some studies suggested that LVI influences the oncological outcome of RCC^13–15,19,21,30^, whereas others did not^16–18,20^.

Most previous studies regarding the prognostic significance of LVI have a few weak points. These analyses were based on relatively small cohorts and did not independently examine those with localized (≤pT2) and locally advanced (≥pT3a) disease, nor did it discriminate among histologic subtypes, even though the prognosis of RCC patients varies according to stage and histologic subtype. The positive rate of LVI in previous studies was relatively high (11.0% in Katz *et al*.^18^, 14.3% in Belsante *et al*.^13^, 18.6% in Kroeger *et al*.^30^) compared to the current study, which may be due to the heterogeneity in the previous studies, especially since they included patients with advanced stage RCC and positive nodes which increased the LVI detection rate.

A relatively large study (n = 833) conducted by Sorbellini^21^ suggested that LVI was associated with recurrence in clear cell RCC patients. Although Sorbellini’s study analyzed a relatively large sample cohort of clear cell RCC, disease recurrence was noted only in 72 patients, including those with advanced RCC. A recently reported study in patients with organ-confined RCC^13^ suggested that LVI is an independent predictor of recurrence and disease-specific survival. This study showed only 15 cases of recurrence in the pT1-2 patient group (n = 333). This small number led to a broader confidence interval (from 1.7 to 92.7), and consequently statistical reliability was not high.

In the current study with a large event size (230 cases of recurrence among patients with or without LVI), unadjusted analysis showed that LVI was a significant prognostic factor for recurrence. However, after adjusting for confounding variables by multivariate Cox regression and propensity score matching, LVI was not a significant prognostic factor for recurrence in localized clear cell RCC. Our hazard ratios show a narrow CI width (Table 3) in comparison with the previous study^13^.

LVI was associated with worse pathologic features, so the positive association between LVI and recurrence in univariate analysis might be due to overall pathologic features. Patients with worse pathology (larger tumor size, higher grade, and sarcomatoid differentiation) were more likely to show positive LVI.

The current study has several strengths and weaknesses. This study is a non-randomized, retrospective study, which inherently could not be representative of general RCC patients. Our study was analyzed without central pathology review despite the retrospective multi-institutional nature of the study. Nonetheless, it has included a large number of localized clear cell RCC patients retrieved from an observational longitudinal multi-institutional database of Korean RCC patients. Moreover, our study was conducted through different statistical models. First, Cox proportional hazard regression analysis was performed to evaluate the relationship between recurrence and CI or LVI. In multivariate Cox proportional hazard regression analysis, the hazard ratio was calculated with or without adjusting confounding variables. Second, propensity score matched analysis was performed. In the propensity score matched analysis, the uneven distribution of known prognostic factors including tumor stage, Fuhrman’s grade, and sarcomatoid differentiation were balanced between the two groups (with or without CI or LVI). On the other hand, one disadvantage of propensity score matching is that it only accounts for observed covariates^31^. To compensate for this disadvantage and to assess the relationships between other confounding variables, the adjusted hazard ratio was calculated by combining propensity score matched analysis. These complementary statistical methods provide solid evidence for the prognostic significance of CI and LVI.

## Conclusion

After adjusting for confounding variables with statistical models using Cox regression and propensity score matching analysis, this study showed that RCI in localized clear cell RCC can provide clinically important prognostic information based on the association between RCI and RCC recurrence. On the other hand, LVI did not influence the recurrence of localized clear cell RCC following curative surgery.
