# Publisher Correction: Renal capsular invasion is a prognostic biomarker in localized clear cell renal cell carcinoma

**DOI:** 10.1038/s41598-018-25182-5

**Published:** 2018-05-11

**Authors:** U-Syn Ha, Kyu Won Lee, Jin-hyung Jung, Seok-Soo Byun, Cheol Kwak, Jinsoo Chung, Eu Chang Hwang, Yong-June Kim, Tae Gyun Kwon, Seok Ho Kang, Sung-Hoo Hong

**Affiliations:** 1 Department of Urology, College of Medicine, The Catholic University of Korea, Seoul, Republic of Korea; 2 Biostatistics, Biomedicine & Health Sciences, College of Medicine, The Catholic University of Korea, Seoul, Republic of Korea; 3 Department of Urology, Seoul National University Bundang Hospital, Seongnam, Republic of Korea; 4 Department of Urology, Seoul National University College of Medicine, Seoul, Korea; 5 Department of Urology, Center for Prostate Cancer, National Cancer Center, Goyang, Korea; 6 Department of Urology, Chonnam National University Medical School, Gwangju, Korea; 7 Department of Urology, Chungbuk National University College of Medicine, Cheongju, Korea; 8 Department of Urology, Kyungpook National University School of Medicine, Daegu, Korea; 9 Department of Urology, Korea University School of Medicine, Seoul, Korea

Correction to: *Scientific Reports* 10.1038/s41598-017-18466-9, published online 09 January 2018

In the original version of this Article, Seok Ho Kang was incorrectly listed as the corresponding author. The correct corresponding author for this Article is Sung-Hoo Hong. Correspondence and request for materials should be addressed to toomey@catholic.ac.kr. This error has now been corrected in the HTML and PDF versions of the Article.

